# Death Certificate–Based ICD-10 Diagnosis Codes for COVID-19 Mortality Surveillance — United States, January–December 2020

**DOI:** 10.15585/mmwr.mm7014e2

**Published:** 2021-04-09

**Authors:** Adi V. Gundlapalli, Amy M. Lavery, Tegan K. Boehmer, Michael J. Beach, Henry T. Walke, Paul D. Sutton, Robert N. Anderson

**Affiliations:** ^1^CDC COVID-19 Response Team, ^2^National Center for Health Statistics, CDC.

Approximately 375,000 deaths during 2020 were attributed to COVID-19 on death certificates reported to CDC (*1*). Concerns have been raised that some deaths are being improperly attributed to COVID-19 (*2*). Analysis of *International Classification of Diseases, Tenth Revision* (ICD-10) diagnoses on official death certificates might provide an expedient and efficient method to demonstrate whether reported COVID-19 deaths are being overestimated. CDC assessed documentation of diagnoses co-occurring with an ICD-10 code for COVID-19 (U07.1) on U.S. death certificates from 2020 that had been reported to CDC as of February 22, 2021. Among 378,048 death certificates listing U07.1, a total of 357,133 (94.5%) had at least one other ICD-10 code; 20,915 (5.5%) had only U07.1. Overall, 97.3% of 357,133 death certificates with at least one other diagnosis (91.9% of all 378,048 death certificates) were noted to have a co-occurring diagnosis that was a plausible chain-of-event condition (e.g., pneumonia or respiratory failure), a significant contributing condition (e.g., hypertension or diabetes), or both. Overall, 70%–80% of death certificates had both a chain-of-event condition and a significant contributing condition or a chain-of-event condition only; this was noted for adults aged 18–84 years, both males and females, persons of all races and ethnicities, those who died in inpatient and outpatient or emergency department settings, and those whose manner of death was listed as natural. These findings support the accuracy of COVID-19 mortality surveillance in the United States using official death certificates. High-quality documentation of co-occurring diagnoses on the death certificate is essential for a comprehensive and authoritative public record. Continued messaging and training (*3*) for professionals who complete death certificates remains important as the pandemic progresses. Accurate mortality surveillance is critical for understanding the impact of variants of SARS-CoV-2, the virus that causes COVID-19, and of COVID-19 vaccination and for guiding public health action.

Death certificates were processed using standard CDC protocols to convert all written text and diagnoses to ICD-10 codes (*4*). Individual data elements were extracted for analysis from death certificates that had the ICD-10 code for COVID-19 (U07.1) listed in Part I (the section for reporting chain of events leading directly to death, the immediate cause of death, and the underlying cause of death) or Part II (the section for reporting all other significant conditions that contributed to death) of the death certificate for deaths that occurred during the calendar year 2020 (Supplementary Figure, https://stacks.cdc.gov/view/cdc/104571) (*3*) and had been reported to CDC by February 22, 2021. First, the location of ICD-10 diagnoses in relation to the COVID-19 diagnosis was used to categorize the co-occurring diagnoses as being in the chain of events that directly caused the death (chain-of-event conditions) or a significant condition contributing to death (significant contributing conditions). Any co-occurring ICD-10 code that appeared on the same line or above U07.1 in Part I was considered to be a chain-of-event condition. Any ICD-10 code that appeared on a line below U07.1 in Part I or in Part II was considered to be a contributing condition. Second, the highest-frequency ICD-10 codes noted in ≥1% of all death certificates with COVID-19 listed in Part I and at least one diagnosis other than COVID-19 were reviewed for consistency and plausibility with conditions known to be associated with severe outcomes in patients with COVID-19 (*5*–*8*). Conditions consistent with those known to be associated with severe COVID-19 outcomes were coded as chain-of-event or significant contributing conditions regardless of their location on the death certificate. For example, a death certificate with an ICD-10 code for respiratory failure listed below U07.1 would be coded as a chain-of-event condition because respiratory failure caused by COVID-19 led directly to the death. Third, less frequently appearing ICD-10 codes that were determined to be consistent with those associated with severe COVID-19 outcomes were also coded as chain-of-event or significant contributing conditions. Finally, death certificates were categorized into five mutually exclusive categories according to the ICD-10 codes recorded on the death certificate 1) only the ICD-10 code for COVID-19; 2) at least one other co-occurring ICD-10 code for a chain-of-event condition; 3) at least one other co-occurring ICD-10 code for a significant contributing condition; 4) an ICD-10 code for both a chain-of-event and significant contributing condition; or 5) an ICD-10 code that could not be categorized as a plausible chain-of-event or significant contributing condition based on current knowledge. Results were stratified by age, sex, race/ethnicity, and setting of death reported on the death certificate. All analyses were conducted using SAS (version 9.4; SAS Institute) and Stata (version 15.0; StataCorp).

Among 378,048 death certificates with the ICD-10 code U07.1, 94.5% (357,133) had at least one other ICD-10 code, whereas 5.5% (20,915) listed only U07.1 (Table 1); 330,198 (87%) listed COVID-19 in Part I. Death certificates with only U07.1 and no other diagnosis accounted for 2.9%–6.6% of death certificates for decedents across all age, sex, and racial/ethnic categories (Table 1). Having only COVID-19 listed on the death certificate was slightly more frequent for death certificates that listed “dead on arrival” (34; 10%) or “decedent’s home” (2,006; 8.6%) as the place of death or “pending” (seven; 13.5%) as the manner of death.

**TABLE 1:** Distribution of death certificates with COVID-19 diagnosis* across five mutually exclusive categories defined by presence and classification of co-occurring diagnoses, by demographic characteristics, setting of death, and manner of death characteristics — National Center for Health Statistics, United States, January–December 2020

Characteristic	No. of death certificates	No. (row %)				
		COVID-19 only	COVID-19 and ≥1 chain-of-event condition only	COVID-19 and ≥1 significant contributing condition only	COVID-19 and ≥1 chain-of-event and ≥1 significant contributing condition	COVID-19 with no plausible chain-of-event or significant contributing condition
**Total**	**378,048**	**20,915 (5.5)**	**128,603 (34.0)**	**67,184 (17.8)**	**151,708 (40.1)**	**9,638 (2.5)**
**Age group, yrs**						
<18	182	8 (4.4)	70 (38.5)	18 (9.9)	22 (12.1)	64 (35.2)
18–29	1,426	77 (5.4)	636 (44.6)	167 (11.7)	401 (28.1)	145 (10.2)
30–39	4,161	275 (6.6)	1,712 (41.1)	550 (13.2)	1,371 (32.9)	253 (6.1)
40–49	11,053	660 (6.0)	4,551 (41.2)	1,408 (12.7)	3,982 (36.0)	452 (4.1)
50–64	55,719	2,911 (5.2)	22,788 (40.9)	6,693 (12.0)	21,666 (38.9)	1,661 (3.0)
65–74	80,705	3,841 (4.8)	30,439 (37.7)	10,756 (13.3)	33,820 (41.9)	1,849 (2.3)
75–84	104,294	5,277 (5.1)	34,784 (33.4)	17,858 (17.1)	44,179 (42.4)	2,196 (2.1)
≥85	120,508	7,866 (6.5)	33,623 (27.9)	29,734 (24.7)	46,267 (38.4)	3,018 (2.5)
**Sex**						
Female	172,615	10,007 (5.8)	55,207 (32.0)	35,525 (20.6)	67,186 (38.9)	4,690 (2.7)
Male	205,423	10,907 (5.3)	73,392 (35.7)	31,658 (15.4)	84,518 (41.1)	4,948 (2.4)
**Race/Ethnicity**						
Hispanic or Latino	70,011	3,680 (5.3)	28,035 (40.0)	7,875 (11.2)	29,016 (41.4)	1,405 (2.0)
American Indian or Alaska Native, non-Hispanic	4,460	266 (6.0)	1,652 (37.0)	501 (11.2)	1,920 (43.0)	121 (2.7)
Asian, non-Hispanic	13,339	676 (5.1)	5,352 (40.1)	1,687 (12.6)	5,331 (40.0)	293 (2.2)
Black, non-Hispanic	59,468	3,114 (5.2)	21,065 (35.4)	9,151 (15.4)	24,688 (41.5)	1,450 (2.4)
Native Hawaiian or Pacific Islander, non-Hispanic	679	20 (2.9)	224 (33.0)	73 (10.8)	350 (51.5)	12 (1.8)
White, non-Hispanic	227,387	12,961 (5.7)	71,227 (31.3)	47,531 (20.9)	89,379 (39.3)	6,289 (2.8)
Multiracial, non-Hispanic	1,123	43 (3.8)	379 (33.7)	165 (14.7)	512 (45.6)	24 (2.1)
Unknown	1,581	155 (9.8)	669 (42.3)	201 (12.7)	512 (32.4)	44 (2.8)
**Setting of death**						
Inpatient	240,770	10,084 (4.2)	103,475 (43.0)	18,719 (7.8)	104,250 (43.3)	4,242 (1.8)
Outpatient/Emergency department	12,851	830 (6.5)	4,287 (33.4)	2,411 (18.8)	4,971 (38.7)	352 (2.7)
Dead on arrival	339	34 (10.0)	98 (28.9)	70 (20.6)	122 (36.0)	15 (4.4)
Decedent's home	23,455	2,006 (8.6)	3,634 (15.5)	8,977 (38.3)	7,579 (32.3)	1,259 (5.4)
Hospice facility	10,458	412 (3.9)	2,722 (26.0)	2,430 (23.2)	4,612 (44.1)	282 (2.7)
Nursing home/Long-term care facility	82,843	6,986 (8.4)	13,183 (15.9)	31,904 (38.5)	27,597 (33.3)	3,173 (3.8)
Other	7,163	549 (7.7)	1,170 (16.3)	2,612 (36.5)	2,522 (35.2)	310 (4.3)
Unknown	169	14 (8.3)	34 (20.1)	61 (36.1)	55 (32.5)	5 (3.0)
**Manner of death**						
Accidental	2,080	0 (—)	233 (11.2)	890 (42.8)	540 (26.0)	417 (20.0)
Could not determine	68	1 (1.5)	16 (23.5)	14 (20.6)	19 (27.9)	18 (26.5)
Homicide	32	0 (—)	6 (18.8)	6 (18.8)	6 (18.8)	14 (43.8)
Natural	344,307	20,399 (5.9)	116,812 (33.9)	63,981 (18.6)	134,267 (39.0)	8,848 (2.6)
Pending investigation	52	7 (13.5)	7 (13.5)	9 (17.3)	28 (53.8)	1 (1.9)
Self-inflicted	61	0 (—)	8 (13.1)	10 (16.4)	4 (6.6)	39 (63.9)
Missing	31,448	508 (1.6)	11,521 (36.6)	2,274 (7.2)	16,844 (53.6)	301 (1.0)

* *International Classification of Diseases, Tenth Revision* code U07.1.

Overall, 97.3% of 357,133 death certificates with at least one other diagnosis (91.9% of all 378,048 death certificates) were noted to have a co-occurring diagnosis that was a plausible chain-of-event condition (e.g., pneumonia, respiratory failure, adult respiratory distress syndrome, cardiac arrest, or sepsis), or significant contributing condition (e.g., hypertension, diabetes, dementia, or chronic obstructive pulmonary disease) (*5*), or both. The most frequent chain-of-event ICD-10 diagnosis codes on 330,198 death certificates that listed COVID-19 on Part I of the death certificate were J18.9 (pneumonia) (45%) and J96.0 (acute respiratory failure) (20%) (Table 2); the most frequent significant contributing condition ICD-10 codes were I10 (essential hypertension) (18%) and E14.9 (diabetes mellitus) (10%). Nearly 75% of all death certificates had a chain-of-event condition, alone or in combination with a significant contributing condition; this finding was noted for adults aged 18–84 years, males and females, persons of all races and ethnicities, those who died in inpatient and outpatient or emergency department settings, and those whose manner of death was listed as natural (Table 1).

**TABLE 2:** Highest-frequency *International Classification of Diseases, Tenth Revision* (ICD-10) codes listed in death certificates with COVID-19 in Part I of death certificate and at least one diagnosis other than COVID-19 (330,198) — National Center for Health Statistics, United States, January–December 2020

Condition (ICD-10 code)	No. (% of 330,198*)
**Conditions listed as chain-of-event conditions on ≥1% of death certificates^†^**	
Pneumonia, unspecified (J18.9)	148,530 (45.0)
Acute respiratory failure (J96.0)	66,609 (20.2)
Respiratory failure, unspecified (J96.9)	47,045 (14.2)
Cardiac arrest, unspecified (I46.9)	36,983 (11.2)
Adult respiratory distress syndrome (J80)	36,297 (11.0)
Sepsis, unspecified (A41.9)	20,117 (6.1)
Viral pneumonia, unspecified (J12.9)	12,421 (3.8)
Asphyxia (R09.0)	10,641 (3.2)
Respiratory arrest (R09.2)	7,009 (2.1)
**Conditions listed as significant contributing conditions on ≥1% of death certificates^§^**	
Essential (primary) hypertension (I10)	58,930 (17.8)
Unspecified diabetes mellitus without complications (E14.9)	34,038 (10.3)
Unspecified dementia (F03)	32,189 (9.7)
Chronic obstructive pulmonary disease, unspecified (J44.9)	24,678 (7.5)
Atherosclerotic heart disease (I25.1)	22,162 (6.7)
Type 2 diabetes mellitus without complications (E11.9)	21,038 (6.4)
Atrial fibrillation and flutter (I48)	19,784 (6.0)
Congestive heart failure (I50.0)	16,841 (5.1)
Tobacco use (F17.9)	16,424 (5.0)
Chronic kidney disease, unspecified (N18.9)	14,525 (4.4)
Alzheimer disease, unspecified (G30.9)	11,220 (3.4)
Hypertensive heart disease without (congestive) heart failure (I11.9)	9,881 (3.0)
Hyperlipidemia, unspecified (E78.5)	9,330 (2.8)
Other specified disorders of kidney and ureter (N28.8)	8,958 (2.7)
Obesity, unspecified (E66.9)	8,913 (2.7)
Chronic kidney disease, stage 5 (N18.5)	7,955 (2.4)
Stroke, not specified as hemorrhage or infarction (I64)	6,494 (2.0)
Heart failure, unspecified (I50.9)	6,436 (1.9)
**Conditions listed on ≥0.5% of death certificates and not identified as known chain-of-event conditions or significant contributing conditions^¶^**	
Other specified general symptoms and signs (R68.8)	1,124 (0.3)
Other lack of normal physiologic development, underweight (R62.8)	734 (0.2)
Dyspnea (R06.0)	363 (0.1)
Senility (R54)	331 (0.1)
Other and unspecified infectious disease (B99.9)	247 (0.1)
Gastrointestinal hemorrhage (K92.2)	213 (0.1)
Unspecified protein energy deficiency (E46)	204 (0.1)
Influenza due to unidentified influenza virus (J11.1)	174 (0.1)
Urinary tract infection (N39.0)	150 (0.0)
Malaise and fatigue (R53)	145 (0.0)
Unspecified fall (W19)	139 (0.0)
Multiple sclerosis (G35)	124 (0.0)

* A total of 330,198 death certificates had COVID-19 listed in Part I of death certificate and at least one other diagnosis listed on the death certificate.

^†^ COVID-19 diagnosis listed in Part I of the death certificate; chain-of-event conditions listed on the same line or above the COVID-19 diagnosis in Part I of the death certificate.

**^§^** COVID-19 diagnosis listed in Part I of the death certificate; significant contributing conditions listed below the COVID-19 line in Part I or in Part II of the death certificate.

**^¶^** COVID-19 diagnosis listed in Part I of the death certificate; co-occurring conditions listed anywhere on the death certificate and not identified as known chain-of-event or significant contributing conditions.

Nearly 18% of death certificates had a co-occurring significant contributing condition only (Table 1). This finding was more frequent for death certificates indicating that the death occurred in the decedent’s home (38.3%), a nursing home or long-term care facility (38.5%), or hospice facility (23.2%). A small proportion (9,638; 2.5%) of death certificates had co-occurring diagnosis codes that could not be plausibly categorized as either a chain-of-event or significant contributing condition. This finding was more frequent among decedents aged <18 years (64; 35.2%) and 18–29 years (145; 10.2%); these age groups represented only 0.4% (1,608) of all death certificates. This was recorded more frequently among decedents who died at home (1,259; 5.4%), were declared “dead on arrival” (15; 4.4%), or whose manner of death was self-inflicted (39; 63.9%), homicide (13; 43.8%), “could not determine” (18; 26.5%), or accidental (417; 20.0%).

Deaths reported from inpatient settings accounted for 240,770 (64%) of all death certificates; 86% of these had co-occurring diagnoses identified as chain-of-event and significant contributing conditions (104,250; 43%) or chain-of-event conditions only (103,475; 43%). A higher proportion of deaths reported from nursing homes or long-term care facilities (22% of all death certificates) listed contributing conditions only (39%) on the death certificate. Contributing conditions were also noted in a larger proportion of death certificates listing the decedent’s home as the location of death (38%); these death certificates were less likely to have co-occurring chain-of-event diagnoses listed on the death certificate.

## Discussion

Among death certificates from calendar year 2020 listing COVID-19 and at least one other co-occurring diagnosis, the documentation is consistent with these deaths being attributable to COVID-19. Specifically, in 97% of 357,133 death certificates with COVID-19 and at least one other diagnosis, the documented chain-of-event and significant contributing conditions were consistent with those reported in clinical and epidemiologic studies to occur among patients with severe COVID-19–associated outcomes (*5*,*9*). Only 5.5% of death certificates had COVID-19 without any other conditions listed. Attributability of death to COVID-19 could not be evaluated for these death certificates and represents an opportunity for improvement in documentation.

A small proportion (2.5%) of death certificates documented conditions that have not currently been described to be associated with COVID-19 critical illness or death. This was noted more often among those who died at home, declared dead on arrival, and whose manner of death was not natural. In particular, a higher percentage of decedents aged <18 years (35.2%) and 18–29 years (10.2%) did not have a chain-of-event or significant contributing condition listed on the death certificate, even though their death certificates did have at least one other diagnosis code along with COVID-19 in Part I or II. Although these age categories constituted a very small proportion of the entire decedent group, the information on the death certificate provides a starting point for identification of other conditions that might contribute to mortality in younger persons. Detailed evaluation of death certificates might provide insights into rare and lesser known conditions that are not yet understood to be associated with or contribute to death from COVID-19.

The findings in this report are subject to at least two limitations. First, the accuracy of documentation of chain-of-event and significant contributing conditions on death certificates is known to be suboptimal (*10*); the effect of COVID-19 on completion of death certificates merits further study, with an emphasis on variation by time, jurisdiction in which the death occurred, age group, race, ethnicity, and setting of death. Second, CDC was unable to compare death certificate data with decedent’s medical records or autopsy reports for end-of-life events and co-occurring diagnoses. Medical record review is needed to confirm findings from this study and elucidate more information for decedents with only COVID-19 listed on their death certificate or those that could not be plausibly categorized as attributable to COVID-19 based on death certificate data alone.

These findings support the accuracy of COVID-19 mortality surveillance in the United States using official death certificates. High-quality documentation of co-occurring diagnoses on the death certificate is essential for a comprehensive and authoritative public record. Continued messaging to and training of professionals who complete death certificates (*3*) remains important as the pandemic progresses. Accurate mortality surveillance is critical for understanding the impact of SARS-CoV-2 variants and of COVID-19 vaccinations and for guiding public health action.

**Summary**

**What is already known about this topic?**

During 2020, approximately 375,000 U.S. deaths were attributed to COVID-19.

**What is added by this report?**

Among 378,048 death certificates from 2020 listing COVID-19, 5.5% listed COVID-19 without codes for any other conditions. Among 357,133 death certificates with at least one other condition, 97% had a co-occurring diagnosis of a plausible chain-of-event condition (e.g., pneumonia or respiratory failure), or a significant contributing condition (e.g., hypertension or diabetes), or both.

**What are the implications for public health practice?**

These findings support the accuracy of COVID-19 mortality surveillance in the United States using official death certificates. High-quality documentation of death certificate diagnoses is essential for an authoritative public record.
